# Comparison of Fourteen Reference Evapotranspiration Models With Lysimeter Measurements at a Site in the Humid Alpine Meadow, Northeastern Qinghai-Tibetan Plateau

**DOI:** 10.3389/fpls.2022.854196

**Published:** 2022-04-27

**Authors:** Licong Dai, Ruiyu Fu, Zhihui Zhao, Xiaowei Guo, Yangong Du, Zhongmin Hu, Guangmin Cao

**Affiliations:** ^1^College of Ecology and Environment, Hainan University, Haikou, China; ^2^Hainan Academy of Forestry, Haikou, China; ^3^Qinghai Provincial Key Laboratory of Restoration Ecology for Cold Region, Northwest Institute of Plateau Biology, Chinese Academy of Sciences, Xining, China

**Keywords:** reference evapotranspiration, alpine meadow, lysimeter measurement, combination models, radiation-based models, temperature-based models

## Abstract

Evapotranspiration is a key component in the terrestrial water cycle, and accurate evapotranspiration estimates are critical for water irrigation management. Although many applicable evapotranspiration models have been developed, they are largely focused on low-altitude regions, with less attention given to alpine ecosystems. In this study, we evaluated the performance of fourteen reference evapotranspiration (ET_0_) models by comparison with large weight lysimeter measurements. Specifically, we used the Bowen ratio energy balance method (BREB), three combination models, seven radiation-based models, and three temperature-based models based on data from June 2017 to December 2018 in a humid alpine meadow in the northeastern Qinghai–Tibetan Plateau. The daily actual evapotranspiration (ET_a_) data were obtained using large weighing lysimeters located in an alpine *Kobresia* meadow. We found that the performance of the fourteen ET_0_ models, ranked on the basis of their root mean square error (RMSE), decreased in the following order: BREB > Priestley-Taylor (PT) > DeBruin-Keijman (DK) > 1963 Penman > FAO-24 Penman > FAO-56 Penman–Monteith > IRMAK1 > Makkink (1957) > Makkink (1967) > Makkink > IRMAK2 > Hargreaves (HAR) > Hargreaves1 (HAR1) > Hargreaves2 (HAR2). For the combination models, the FAO-24 Penman model yielded the highest correlation (0.77), followed by 1963 Penman (0.75) and FAO-56 PM (0.76). For radiation-based models, PT and DK obtained the highest correlation (0.80), followed by Makkink (1967) (0.69), Makkink (1957) (0.69), IRMAK1 (0.66), and IRMAK2 (0.62). For temperature-based models, the HAR model yielded the highest correlation (0.62), HAR1, and HAR2 obtained the same correlation (0.59). Overall, the BREB performed best, with RMSEs of 0.98, followed by combination models (ranging from 1.19 to 1.27 mm day^−1^ and averaging 1.22 mm day^−1^), radiation-based models (ranging from 1.02 to 1.42 mm day^−1^ and averaging 1.27 mm day^−1^), and temperature-based models (ranging from 1.47 to 1.48 mm day^−1^ and averaging 1.47 mm day^−1^). Furthermore, all models tended to underestimate the measured ET_a_ during periods of high evaporative demand (i.e., growing season) and overestimated measured ET_a_ during low evaporative demand (i.e., nongrowing season). Our results provide new insights into the accurate assessment of evapotranspiration in humid alpine meadows in the northeastern Qinghai–Tibetan Plateau.

## Introduction

Evapotranspiration is a key parameter in the simultaneous processes of heat and water transfer to the atmosphere *via* transpiration and evaporation in the soil–plant–atmosphere system (Sentelhas et al., 2010), thereby playing an important role in water balance calculations, water allocation, and water irrigation management. Thus, accurate estimates of evapotranspiration could improve water management strategies and promote efficient water resource use, especially in regions with water shortages (Sun et al., 2011).

To date, direct evapotranspiration measurements have been achieved by a variety of methods, such as the Bowen ratio energy balance (BREB) system (Irmak et al., 2003, 2005; Si et al., 2005; Irmak and Irmak, 2008), lysimeters (Jia et al., 2006; Valipour, 2015), and the eddy covariance technique (Novick et al., 2009; Zhang et al., 2018). Alternatively, evapotranspiration can be indirectly assessed by applying various reference evapotranspiration (ET_0_) equations. Several ET_0_ models have been widely used for evapotranspiration calculation and can be classified into three types, namely, radiation-based models (Hargreaves and Samani, 1985), temperature-based models (Trajkovic et al., 2019), and combination models (Penman, 1963). While the development of these models has undoubtedly benefited the calculation of evapotranspiration, it remains difficult to choose the optimal one because of the availability of observed data, and most models have not been evaluated against lysimeter measurements across a range of regions and climates (Liu et al., 2017; Kiefer et al., 2019). To select the best-performing models, many studies have been conducted to assess model performance under various climates. For instance, the Food and Agriculture Organization of the United Nations (FAO) recommends the Penman–Monteith FAO-56 (PM-56) as the standard equation for estimating models ET_0_ (Allen et al., 1998), and this has been widely used worldwide when compared with other equations (Cai et al., 2007). The advantage of the PM equation is that it does not require any local calibration because it incorporates both physiological and aerodynamic parameters, and it has been well tested based on lysimeter data (Trajkovic, 2009).

Although many models have been widely used to estimate ET, most previous models have only been evaluated with respect to PM-56 (Cao et al., 2015; Liu et al., 2017), with few being tested against lysimeter measurements. Furthermore, the application of the PM-56 equation requires many meteorological inputs, such as wind speed, temperature, humidity, and solar radiation, which are often not available in regions with harsh environments (Tabari et al., 2012; Martel et al., 2018). Thus, it is essential to develop a relatively accurate ET_0_ model that requires fewer meteorological parameters to allow more simplified estimates of evapotranspiration than those of PM-56, applicable across a range of climatic conditions (Tabari and Talaee, 2011). For example, Tabari (2010) assessed four ET_0_ models in an arid climate and found that the Turc model performed the best. Meanwhile, the Hargreaves equation performed best in semiarid regions (Sabziparvar and Tabari, 2010). Liu et al. (2017) compared sixteen models for ET_0_ against weighing lysimeter measurements and found that the combination models performed best for estimating evapotranspiration in semiarid regions. Overall, most previous studies have been conducted in low-humidity conditions at low altitudes (i.e., arid and semiarid regions) (Sentelhas et al., 2010; Liu et al., 2017), with few studies in humid climates, particularly in alpine ecosystems. Compared with arid and semiarid regions in low altitudes, the interaction soil-plant-atmosphere condition change in the humid alpine meadow was more affected by net radiance (Dai et al., 2021), thus, the ET_0_ models in arid and semiarid regions might not be suitable for humid alpine meadow. It was urgent to improve the accuracy of evapotranspiration observations in alpine regions by comparing reference evapotranspiration models with lysimeter measurements in the humid alpine meadow.

The Qinghai–Tibetan Plateau (QTP), with an average altitude of 4,000 m, is the world's highest alpine ecosystem and is also known as the “Asian tower,” playing an important role in ensuring the safety of water resources in China and southeast Asia (Zou et al., 2017; Dai et al., 2019b). Furthermore, the permafrost and seasonally frozen ground account for 50–56% of the total Qinghai-Tibet Plateau area, and the alpine ecosystem was more sensitive to global warming compared with other ecosystems owing to its high altitude (Zou et al., 2017). Therefore, an accurate estimation of evapotranspiration in an alpine ecosystem could not only provide new insights into the water cycle but also benefit the formulation of water resource management strategies. Furthermore, given the uncertainty and confusion in the selection of evapotranspiration equations across different regions and climates, it is critical to thoroughly understand the performance of various models in the humid alpine meadow (Zhang et al., 2018). In this study, we compared fourteen ET_0_ models against lysimeter measurements on the northern Tibetan Plateau, with the aim of selecting the best fit model over a humid alpine meadow on the northeastern QTP to estimate evapotranspiration.

## Materials and Methods

### Study Area

**Figure 1:**
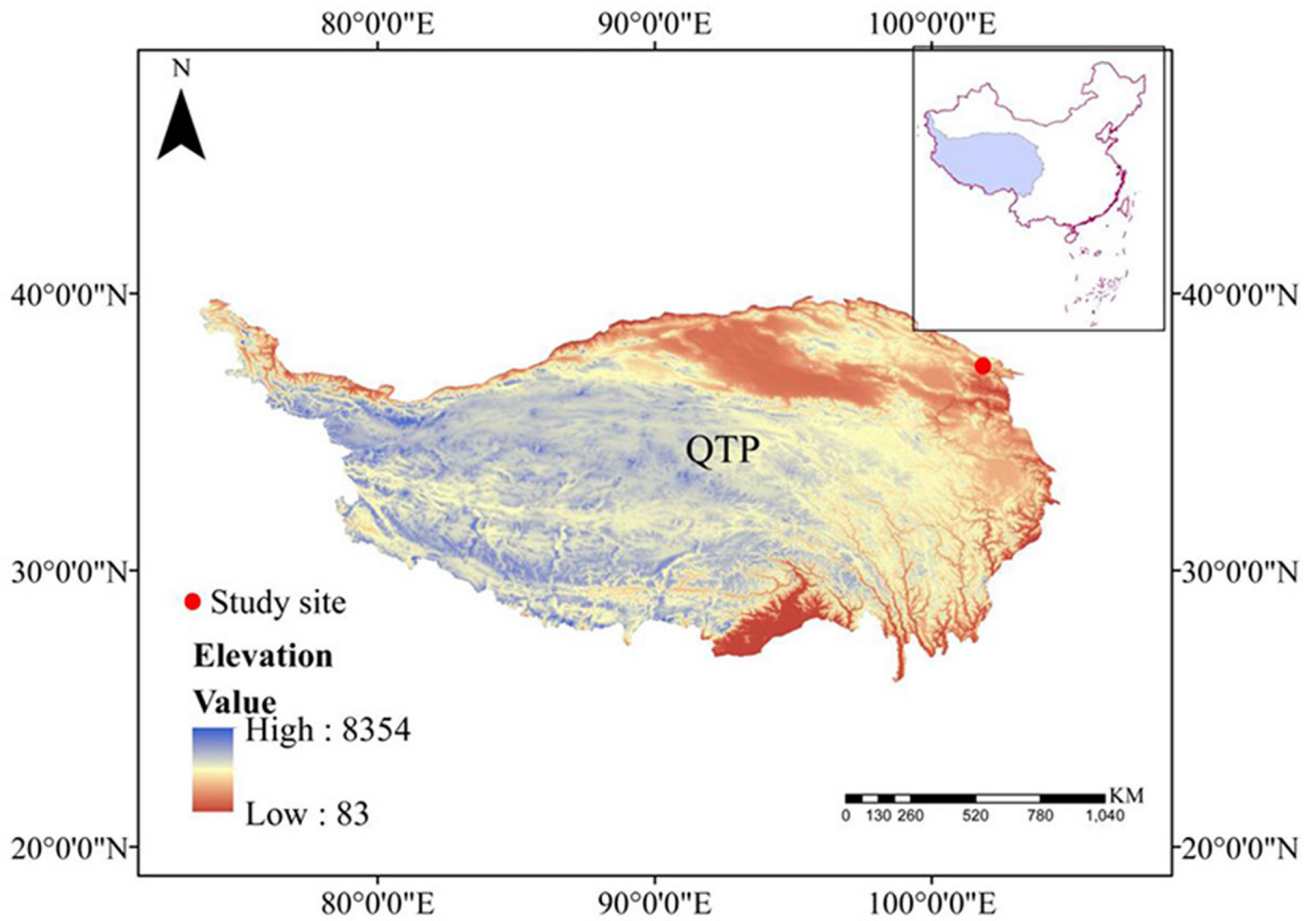
The location of the study area.

This study was conducted at the Haibei National Field Research Station, Qinghai, China (37°37′ N, 101°19′E), which is located on the Northeastern QTP at an elevation of 3,200 m MASL (Figure 1). This area is characterized by a plateau continental monsoon climate, with well-developed seasonally frozen ground. The average annual air temperature is −1.7°C, with the maximum monthly temperature in July (10.1°C) and the minimum monthly temperature in January (−15°C). The annual precipitation is ~580 mm, of which 80% falls in the growing season (i.e., from May to September), leading to high water content (close to field capacity) in the soil during the growing season, thus, the evapotranspiration during the growing season was not limited by soil water content (Dai et al., 2021). The average annual pan evaporation is ~1,191.4 mm (Zhang et al., 2018). The soil type around the lysimeter system is classified as Mat-Gryic Cambisol, which belongs to clay loam, and has a thickness of ~60–80 cm (Dai et al., 2019a), and the basic soil property is shown in Table 1. The grass crop is dominated by perennial sedge and graminoid species, including *Kobresia humilis, Stipa aliena*, and *Elymus nutans*, with ~8–15 cm in height, which together constitutes 60–80% of plant cover around the lysimeter system (Dai et al., 2021).

**Table 1:** The basic soil properties around the lysimeter system.

**Soil depth** **(cm)**	**Clay content** **(%)**	**Silt content** **(%)**	**Sand content** **(%)**	**Soil bulk density** **(g cm^**−3**^)**	**Soil organic matter** **(g kg^**−1**^)**	**Soil porosity** **(%)**	**Soil water content**	**Saturation capacity** **(m^**3**^ m^**−3**^)**	**Field capacity** **(m^**3**^ m^**−3**^)**	**Wilting point** **(m^**3**^ m^**−3**^)**
0–10	8.88	60.03	31.05	0.74	145.53	72.13	0.43	0.63	0.41	0.30
10–20	8.24	67.21	24.52	1.06	72.92	60.02	0.38	0.57	0.35	0.26
20–30	8.70	67.94	23.34	1.04	51.94	60.68	0.36	0.53	0.34	0.26
30–40	8.56	66.43	24.99	1.08	43.15	59.30	0.35	0.51	0.34	0.26
40–50	9.63	68.66	21.69	1.04	42.41	60.62	0.35	0.50	0.33	0.24

### Actual Evapotranspiration Measurement and Data Quality Control

The actual evapotranspiration (ET_a_) was measured using large-scale weighing lysimeters (height 2 m, diameter 1 m, and resolution 10 g) installed in the central alpine *Kobresia* meadow of the Haibei National Field Research Station. Lysimeters are vessels containing both monolites and repacked soils and are embedded completely in the soil at their top level with the soil surface. The topsoil, including the root, was composed of monolites, while deep soil, not including roots, comprised repacked soils. The soil was cut off from the parent soil at the base of the lysimeters, and the lower lysimeter boundary was exposed to atmospheric pressure. Measurements of ET_a_ were based on changes in weight, with measurements at 30-min intervals recorded by a data logger (CR1000, Campbell, USA) and converted to daily values. To ensure data quality, all negative or abnormal ET_a_ values caused by falling soil particles were discarded; outliers that differed more than three times from average ET_a_, which yielded 393 days of data spanning June 2017–December 2018. According to *in situ* phenological observations of the dominant plant foliage, we defined the growing season as the period from May 1 to September 30, while the period from October 1 to April 30 of the following year was defined as the nongrowing season (Zhang et al., 2018).

Given that the soil moisture of the root region was more than the field capacity (Table 1), thus, the ET_a_ in this study was mainly not limited by surface moisture. Furthermore, the plant height was 8–12 cm, actively growing, and completely shading the ground, which satisfies the definition of reference evapotranspiration (defined as 8–15 cm high, uniform, actively growing, green grass that completely shades the soil and not limited by soil water availability), and we, thus, could assume that the ET_a_ in this study could be considered as reference crop evapotranspiration or potential evaporation. To verify the assumption that ET_a_ in this study was not limited by soil water availability, our previous studies showed that the annual Priestley–Taylor coefficient values (ratio of ET_a_ to ET_eq_) ranged from 1.08 to 1.14 (ET_eq_ represents the minimum possible evapotranspiration from a moist surface and depends only on air temperature and available energy), and the annual decoupling coefficient Ω ranged from 0.43 to 0.48 (Zhang et al., 2018), which provided direct evidence that ET_a_ in this study occurred under strongly energy-limited conditions rather than soil water availability constraints.

### Meteorological and Soil Water Content Data Collection

All meteorological variables needed to calculate ET_0_ using various models were obtained or estimated from the weather station at Haibei Station, including precipitation (PPT), relative humidity (RH), wind speed (WS), net radiation (*R*_n_), total radiation (*R*_s_), soil heat flux (*G*), maximum air temperature (*T*_max_), vapor pressure deficit (VPD), minimum air temperature (*T*_min_), and mean air temperature (*T*_a_). PPT was collected using a PPT gauge (52203, RM Young, USA) at 0.5 m height. Radiation was measured by four radiometers (CNR4, Kipp & Zonen, Netherlands) at 1.5 m height; RH, WS, and T were measured at 1.5 m height (HMP45C, Vaisala, Finland), and WS was converted to 2 m height (${\text{u}}_{2}=u_{z}\frac{4.87}{\ln\;\left(67.82-5.42\right)}$) for calculating ET_0_. The *G* was measured using three heat flux plates (HFT-3, Campbell, USA), which were buried 5 cm beneath the surface. Half-hourly means of meteorological data were stored using a data logger (9210 XLITE, Sutron, USA). The BREB method parameter was determined using an eddy covariance system installed near a lysimeter system, which included a three-dimensional ultrasonic anemometer (CSAT3, Campbell, USA) and an open-path infrared CO_2_/H_2_O gas analyzer (LI-7500A, LI-Cor, USA), and 30-min fluxes were calculated and logged with a SMARTFLUX system (LI-COR, USA) (Zhang et al., 2018). The soil water content was measured using the oven-drying method at depths of 0–10, 10–20, 20–30, 30–40, and 40–50 cm through a soil auger in July because the evapotranspiration reaches its maximum in July with its maximum water demand.

### Reference Evapotranspiration Models

The reference evapotranspiration was defined as 8–15 cm high, uniform, actively growing, green grass that completely shades the soil and not limited by soil water availability (Doorenbos and Pruitt, 1977). A total of fourteen often-used ET_0_ models were selected for comparison, including BREB, three combination models (1963 Penman, FAO-24 Penman, and FAO-56 PM), seven radiation models [Priestley–Taylor, DeBruin–Keijman, Makkink, Makkink (1957), Makkink (1967), IRMAK1, and IRMAK2], and three temperature-based models (Hargreaves, Hargreaves1, and Hargreaves2), to compare their performance using the lysimeter measurement. The specific equations and parameters of the models are listed in Table 2.

**Table 2:** Details of selected models for evaluation and input parameters in each model.

**Type**	**Formula**	**Equations (abbreviation)**
Bowen ratio-energy balance method	$\lambda ET=\frac{R_{n}-G}{1+\beta }$ $\beta \text{=}\gamma \frac{\Delta T}{\Delta e}$	BREB
	$ET_{0}=\frac{0.408\Delta \left(R_{n}-G\right)+\gamma \frac{900}{T+273}u_{2}\left(e_{s}-e_{a}\right)}{\Delta +\gamma \left(1+0.34u_{2}\right)}$	FAO56 Penman-Monteith (FAO-56PM)
Combination	$\lambda ET_{0}=c\left[\frac{\Delta }{\Delta +\gamma }\left(R_{n}-G\right)+2.7\frac{\gamma }{\Delta +\gamma }\left(1+0.864u_{2}\right)\left(e_{s}-e_{a}\right)\right]$c = 1	FAO24Penman (FAO-24 Pen)
	$\lambda \;\cdot \text{E}{\text{T}}_{0}=\left[\frac{\Delta }{\Delta +\gamma }\left(R_{n}-G\right)+6.43\frac{\gamma }{\Delta +\gamma }\left(a_{w}+b_{w}u_{2}\right)\left(e_{s}-e_{a}\right)\right]$a_w_=1, b_w_= 0.537	Penman (1963) (Pen-63)
Radiation-based	$\lambda ET_{0}=\alpha \frac{\Delta }{\Delta +\gamma }\left(R_{n}-G\right)$	Priestley-Taylor (Nemani et al.)
	$\lambda ET_{0}=\frac{\Delta }{0.85\Delta +0.63\gamma }\left(R_{n}-G\right)$	De Bruin-Keijman (DK)
	$\lambda ET_{0}=0.63\frac{\Delta }{\Delta +\gamma }R_{s}$	Makkink
	$ET_{0}=0.7\frac{\Delta }{\Delta +\gamma }\frac{R_{s}}{\lambda }$	Makkink (1967)
	$ET_{0}=0.61\frac{\Delta }{\Delta +\gamma }\frac{R_{s}}{\lambda }-0.12$	Makkink (1957)
	*ET*_0_ = −0.611+0.149 × *R*_*s*_+0.079 × *T*	IRMAK1
	*ET*_0_ = −0.642+0.174 × *R*_*s*_+0.0353 × *T*	IRMAK2
Temperature based	$ET_{0}=0.0023{\left(TD\right)}^{0.5}\left(T+17.8\right)R_{a}$	Hargreaves (HAR)
	$ET_{0}=0.408\times 0.0030\left(T+20\right){\left(\Delta T\right)}^{0.4}R_{a}$	Hargreaves1(HAR1)
	$ET_{0}=0.408\times 0.0025\left(T+16.8\right){\left(\Delta T\right)}^{0.5}R_{a}$	Hargreaves2 (HAR2)

*ET_0_, reference crop evapotranspiration (mm day^−1^); ΔT and Δe are the temperature (°C) and vapor pressure (kPa) difference between the two measurement levels, respectively. R_n_, net radiation (MJ m^−2^); G, soil heat flux (MJ m^−2^ day^−1^); Δ, slope of the saturation vapor pressure curve (kPa °C^−1^); γ, psychrometric constant (kPa °C^−1^); u_2_, wind speed at 2 m (m s^−1^); e_s_, saturated vapor pressure (kPa); e_a_, actual vapor pressure (kPa); λ, vaporizing latent of water (MJ kg^−1^); T, mean air temperature (°C); R_s_, total radiation (MJ m^−2^ day^−1^); R_a_, extraterrestrial solar radiation (MJ m^−2^); TD, the difference between T_max_ and T_min_*.

### Evaluation Criteria

In this study, the ET_a_ measured by the large-scale weighing lysimeters and the performances of the ET_0_ models were compared with these lysimeter system estimates on a daily basis. Pairwise comparisons were conducted using a general linear regression. For further comparison, the root means squared error (RMSE), percentage error of estimate (PE), mean absolute error (MAE), and coefficient of determination (*R*^2^) were used to evaluate the ET_0_ models. The RMSE, PE, MAE, and *R*^2^ are defined as follows:


(1) $$\begin{array}{r} RMSE=\sqrt{\frac{\sum_{i=1}^{n}{\left(P_{i}-O_{i}\right)}^{2}}{n}} \end{array}$$

(2) $$\begin{array}{r} PE=|\frac{\bar{P}-\bar{O}}{\bar{O}}|\times 100 \end{array}$$

(3) $$\begin{array}{r} MAE=\frac{\sum_{i=1}^{n}\left(P_{i}-O_{i}\right)}{n} \end{array}$$

(4) $$R^{2}=\frac{{\left[{\sum^{\text{​}}}_{i=1}^{n}(P_{i}-\bar{P})(O_{i}-\bar{O})\right]}^{2}}{{\sum^{\text{​}}}_{i=1}^{n}{\left(P_{i}-\bar{P}\right)}^{2}{\sum^{\text{​}}}_{i=1}^{n}{\left(O_{i}-\bar{O}\right)}^{2}}$$

where *P*_i_ is the predicted value; *O*_i_ is the observed value; $\bar{P}$ and $\bar{O}$ are the averages of *P*_i_ and *O*_i_, respectively; and *n* is the total number of data points.

### Statistical Analysis

To achieve the best comparison between the models and measurements, we need to select the dominant meteorological factors affecting the measured ET_a_. Given that it may not be appropriate to explore results based solely on the coefficient of independent variables in multiple regression analysis, owing to the strong collinearity and nonlinearities among meteorological factors, we adopted a boosted regression tree (BRT) model to quantitatively evaluate the relative influences of meteorological variables on measured ET_a_. BRT is a machine-learning method based on the classification regression tree algorithm (CART). This method generates multiple regression trees through random selection and self-learning methods, which can improve model stability and prediction accuracy. During operation, some data are randomly selected many times to analyze the influence of independent variables on dependent variables and to quantitatively evaluate the relative effect of independent variables on dependent variables, while the remaining data are used to test the fitting results (Elith et al., 2008). Most importantly, the BRT can evaluate the relative influence of an independent variable on a dependent variable, without transformations, and can cope well with nonlinear relationships. Furthermore, the BRT exhibits good performance in dealing with stronger collinearity and nonlinearities. Thus, the BRT was adopted to evaluate the individual influences of the controlling factors on the measured evapotranspiration. All statistical analyses were performed using R software version 3.03 (R Development Core Team, 2006), and all figures were plotted using Origin 9.0.

## Results

### Seasonal Variation in ET_a_ and Environmental Variables

**Figure 2:**
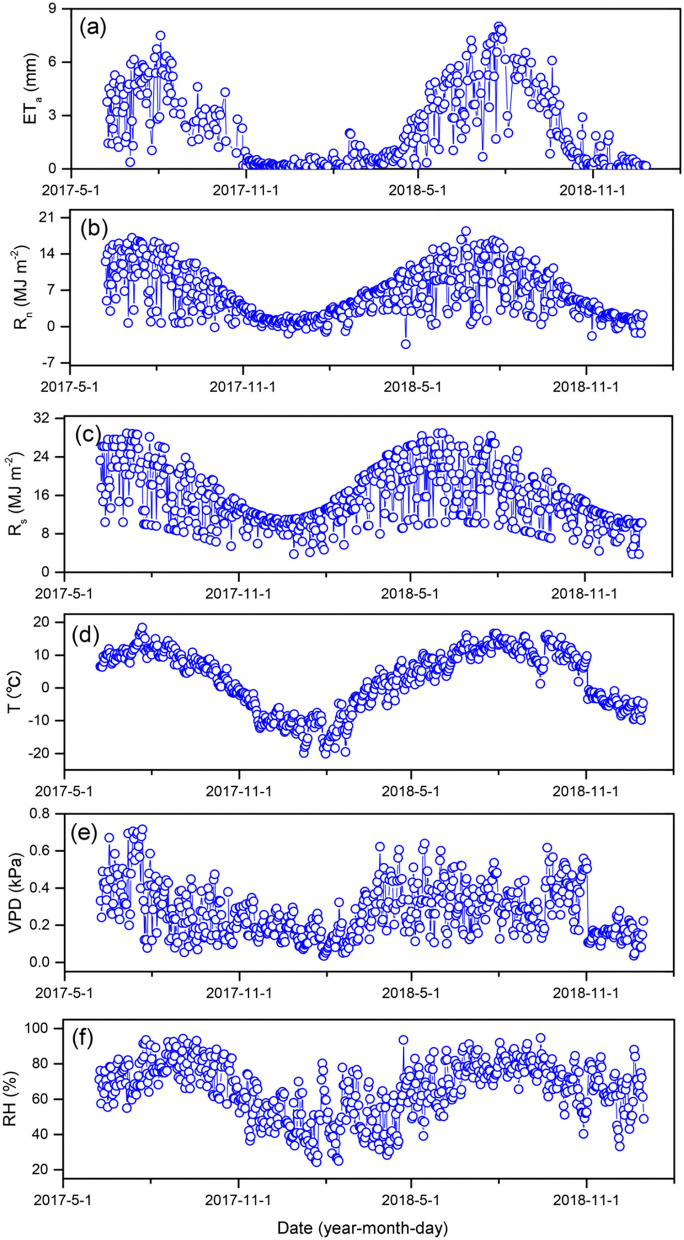
Seasonal variation of actual evapotranspiration (ET_a_) **(a)**, net radiation (R_n_) **(b)**, total radiation (R_s_) **(c)**, mean air temperature (T) **(d)**, vapor pressure deficit (VPD) **(e)**, and relatively humid (RH) **(f)**.

The measured ET_a_ showed a clear seasonal pattern, and the growing season ET_a_ was significantly higher than that in the nongrowing season (*P* < 0.05) (Figure 2a). The average measured daily ET_a_ during the study period was 2.33 mm day^−1^, with an average daily measured ET_a_ of 4.14 and 0.65 mm day^−1^ during the growing season and nongrowing season, respectively (Figure 2a). Environmental variables showed a similar seasonal pattern, with maximum and minimum values in the growing and nongrowing seasons (Figure 2). The average daily *R*_n_, *R*_s_, *T*_a_, VPD, and RH during the growing season were 9.53 MJ m^−2^, 18.50 MJ m^−2^, 10.15°C, 0.33 kPa, and 74.88%, respectively (Figure 2). The average daily *R*_n_, *R*_s_, *T*_a_, VPD, and RH during the nongrowing season were 3.28 MJ m^−2^, 12.65 MJ m^−2^, −3.67°C, 0.23 kPa, and 57.16%, respectively.

### Comparison of Daily Evapotranspiration Between Reference Evapotranspiration Models and Lysimeter Measurements Throughout the Study Period

**Figure 3:**
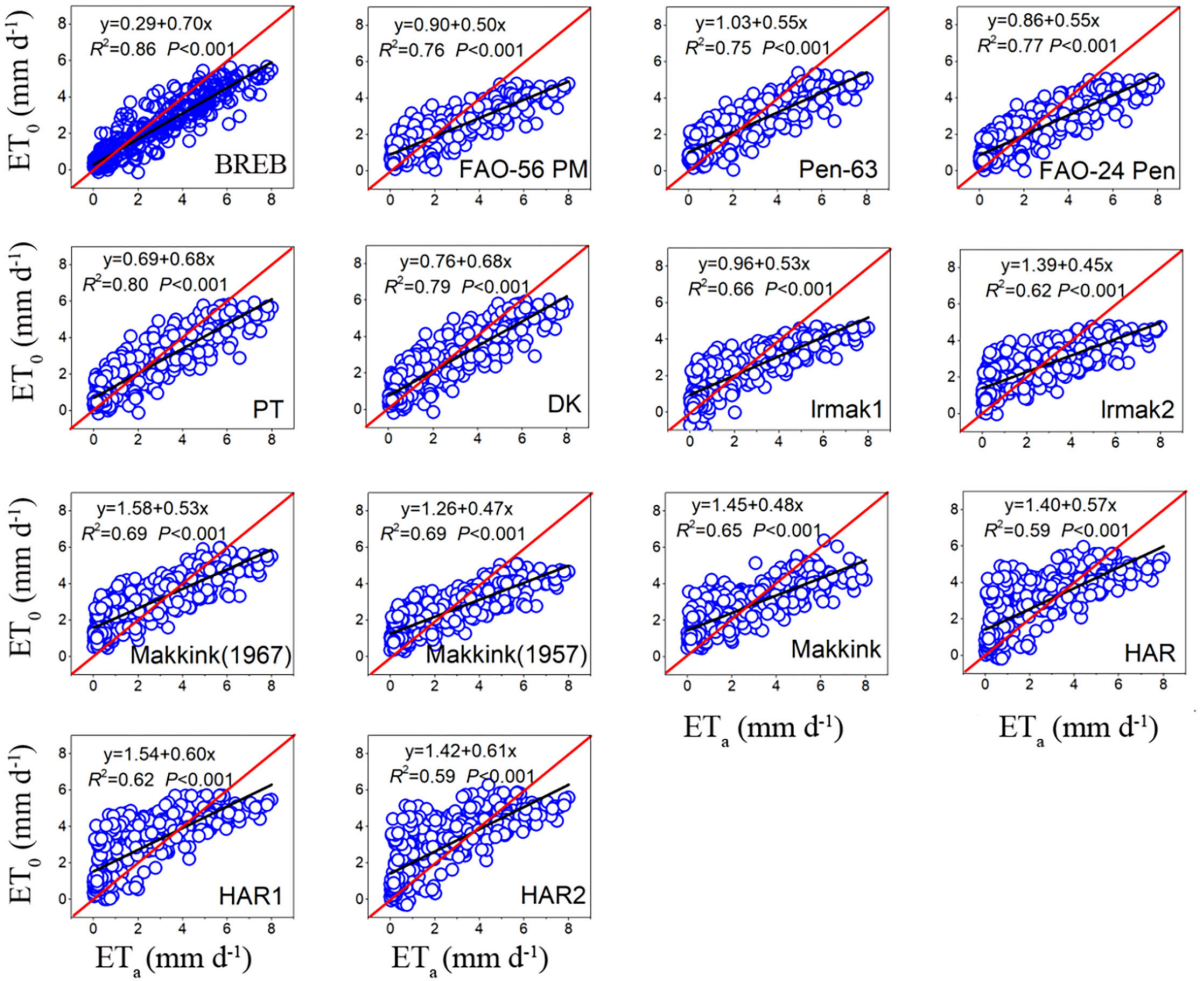
The relationship between daily ET_0_ of model estimates and ET_a_ of lysimeter measurements during the whole study period (data points = 393).

A comparison of fourteen evapotranspiration equations against the lysimeter measurements is presented in Figure 3, Table 3, showing that the relationship between daily ET_0_ calculated by the ET_0_ models and lysimeter measurements ET_a_ was all significant (*P* < 0.01), with high coefficients of determination (*R*^2^) ranging from 0.59 to 0.86. For the combination models, FAO-24 Penman (FAO-24 Pen) yielded the highest correlation (0.77), followed by PM-56 (0.76) and Penman 63 (Pen-63) (0.75). For radiation-based models, PT obtained the highest correlation (0.80), followed by DK (0.79), Makkink (1967) (0.69), Makkink (1957) (0.69), IRMAK1 (0.66), Makkink (0.65), and IRMAK2 (0.62). For temperature-based models, HAR obtained the highest correlation (0.62), and HAR1(0.59) and HAR2 (0.59) obtained the same correlation. The daily estimates of combination models generally underestimated the ET_a_ values measured by lysimeter, with MAEs of −0.26 to −0.01 mm day^−1^. However, the radiation-based models (except PT and IRMAK1) and temperature-based models generally overestimated the ET_a_ values, with MAEs ranging from −0.14 to 0.50 mm day^−1^ for radiation-based models and 0.40–0.59 mm day^−1^ for temperature-based models.

**Table 3:** Summary of statistics for daily ET_0_ between lysimeter measurements and model estimates during the whole study period (data points = 393).

**Model**	**Values of ET**_**0**_ **(mm d**^**−1**^**)**			**Difference in ET**_**0**_			
	**Max**	**Min**	**Mean**	**RMSE (mm d^**−1**^)**	**MAE (mm d^**−1**^)**	**PE (%)**	***R*** **^2^**
Measured	8.00	0.01	2.33				
BREB	5.66	−0.16	1.93	0.98	−0.40	17.13	0.86
FAO-56PM	4.79	0.06	2.07	1.27	−0.26	11.01	0.76
Pen-63	5.38	0.04	2.30	1.19	−0.02	1.00	0.75
FAO-24 Pen	5.05	−0.02	2.14	1.19	−0.19	7.99	0.77
PT	5.93	−0.17	2.27	1.02	−0.06	2.56	0.80
DK	6.02	−0.19	2.34	1.03	0.01	0.57	0.79
Makkink	6.38	0.45	2.57	1.38	0.24	10.27	0.65
Makkink(1967)	5.97	0.47	2.82	1.36	0.50	21.35	0.69
Makkink(1957)	5.08	0.29	2.34	1.34	0.01	0.59	0.69
IRMAK1	4.70	−0.83	2.19	1.32	−0.14	5.86	0.66
IRMAK2	4.81	−0.05	2.43	1.42	0.11	4.62	0.62
HAR	5.95	−0.18	2.73	1.47	0.40	17.37	0.62
HAR1	5.72	−0.01	2.92	1.47	0.59	25.54	0.59
HAR2	6.27	−0.31	2.84	1.48	0.51	22.05	0.59

**Figure 4:**
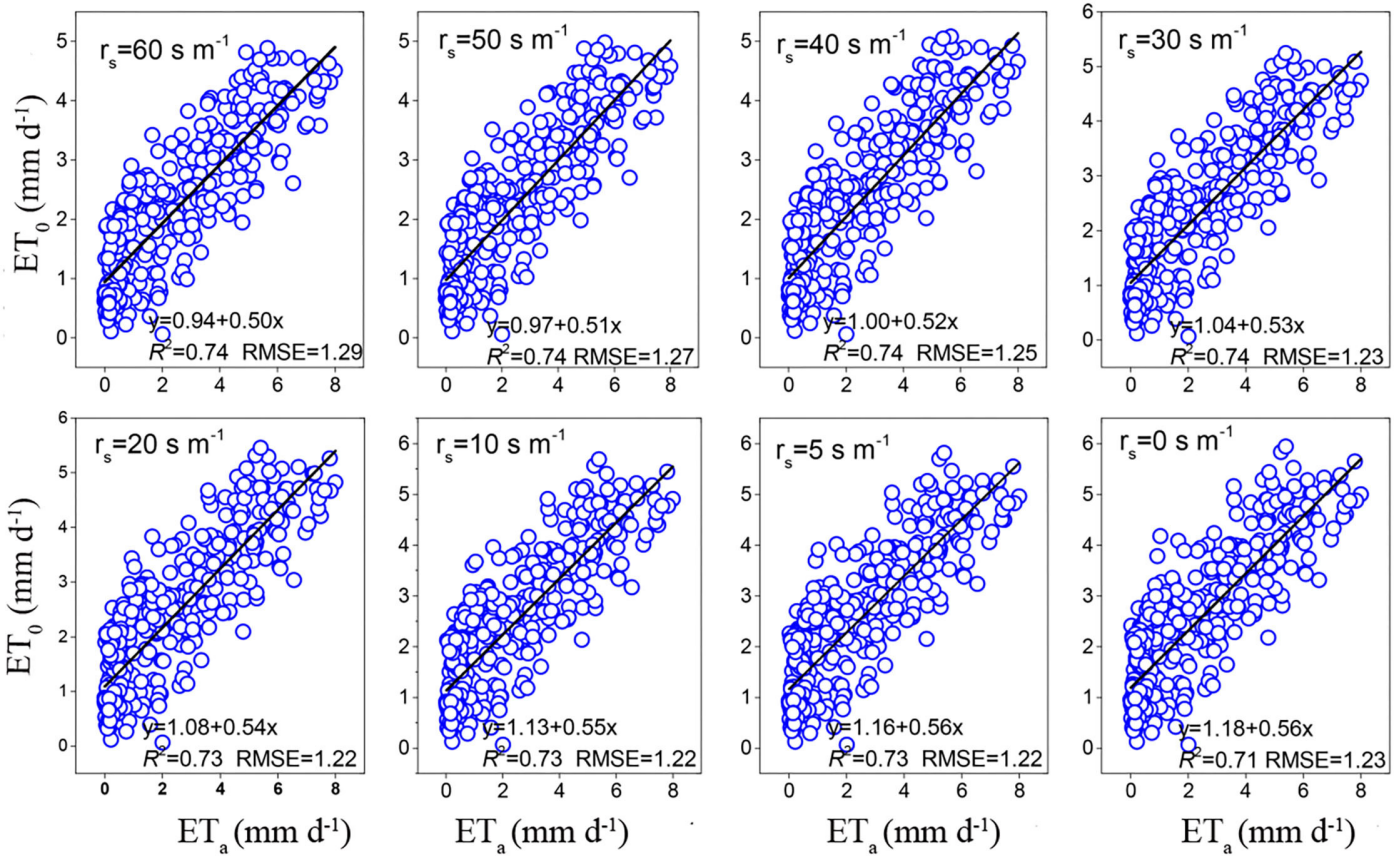
Effect of different surface resistances on daily estimates of the FAO56 Penman–Monteith.

Throughout the study period, the RMSE of the BREB method was 0.98, and the RMSE of combination models ranged from 1.19 to 1.27 mm day^−1^ and averaged 1.22 mm day^−1^. Furthermore, the RMSE of FAO-56 PM increased from 1.22 to 1.29 mm day^−1^ as aerodynamic resistance (*r*_s_) changed from 20 to 60 s m^−1^ (Figure 4). The RMSE for radiation-based models ranged from 1.02 to 1.42 mm day^−1^ and averaged 1.27 mm day^−1^, and the RMSE for temperature-based models ranged from 1.47 to 1.48 mm day^−1^ and averaged 1.47 mm day^−1^. Based on the RMSE, the performance of the fourteen evapotranspiration models decreased in the following order: BREB (0.98) > PT (1.02) > DK (1.03) > Pen-63 (1.19) > FAO-24 Pen (1.19) > PM-56 (1.27) > IRMAK1 (1.32) > Makkink (1957) (1.34) > Makkink (1967) (1.36) > Makkink (1.38) > IRMAK2 (1.42) > HAR (1.47) > HAR1 (1.47) > HAR2 (1.48). The best model was 34% and 30% more accurate than the poorest (HAR2) and the commonly used FAO-56 PM equation, respectively. Furthermore, Pen-63 and FAO-24 Pen demonstrated better performance than the commonly used PM-56 equation. Overall, for the entire study period, the BREB yielded the best performance, followed by the combination, radiation-based, and temperature-based models.

### Comparison of Daily Evapotranspiration Between Reference Evapotranspiration Models and Lysimeter Measurements During the Growing Season

**Figure 5:**
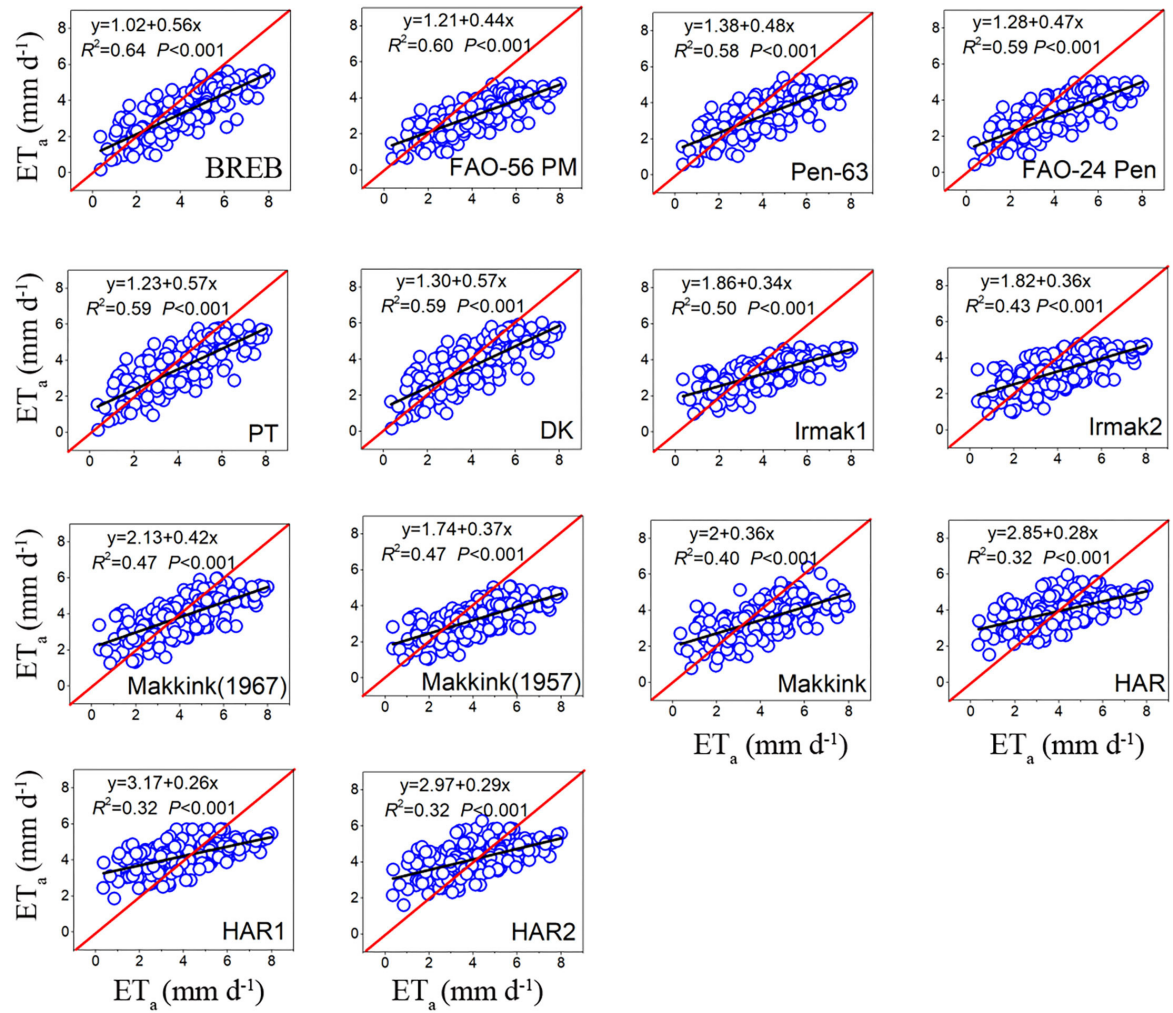
The relationship between daily ET_0_ of model estimates and ET_a_ of lysimeter measurements during the growing season (data points = 181).

During the growing season, the daily ET_0_ calculated by fourteen evapotranspiration models was significantly correlated with the lysimeter measurements ET_a_ (*P* < 0.01), with *R*^2^ ranging from 0.32 to 0.64 (Figure 5, Table 4). Of the combination models, PM-56 had the highest *R*^2^ (0.60), followed by FAO-24 Pen (0.59) and Pen-63 (0.58). Of the radiation-based models, PT and DK obtained the highest *R*^2^ (0.59), followed by IRMAK1 (0.50), Makkink (1967) (0.47), Makkink (1957) (0.47), IRMAK2 (0.43), and Makkink (0.40). Notably, Makkink (1967) and Makkink (1957) had the same *R*^2^ (0.47). Of the temperature-based models, HAR, HAR1, and HAR2 obtained the same *R*^2^ values (0.32). Interestingly, all models (except HAR1 and HAR2) generally underestimated ET_a_ during the growing season, values of MAE ranged from −1.10 to −0.15 mm day^−1^, with PM-56 having the largest underestimation (26.59%) and HAR the minimum underestimation (3.56%) (Table 4).

**Table 4:** Summary of statistics for daily ET_0_ between lysimeter measurements and model estimates during the growing season (data points = 189).

**Model**	**Values of ET**_**0**_ **(mm d**^**−1**^**)**			**Difference in ET**_**0**_			
	**Max**	**Min**	**Mean**	**RMSE (mm d^**−1**^)**	**MAE (mm d^**−1**^)**	**PE (%)**	***R*** **^2^**
Measured	8.00	0.35	4.14				
BREB	5.66	0.16	3.34	1.31	−0.79	19.17	0.64
FAO-56PM	4.79	0.62	3.04	1.58	−1.10	26.59	0.60
Pen-63	5.38	0.57	3.36	1.38	−0.78	18.86	0.58
FAO-24 Pen	5.05	0.43	3.21	1.46	−0.93	22.48	0.59
PT	5.93	0.14	3.59	1.22	−0.55	13.31	0.59
DK	6.02	0.14	3.67	1.19	−0.47	11.41	0.59
Makkink	6.38	0.79	3.51	1.47	−0.63	15.13	0.40
Makkink(1967)	5.97	1.27	3.87	1.28	−0.27	6.45	0.47
Makkink(1957)	5.08	0.99	3.25	1.55	−0.88	21.37	0.47
IRMAK1	4.70	0.99	3.27	1.54	−0.87	20.95	0.50
IRMAK2	4.81	0.89	3.30	1.55	−0.84	20.29	0.43
HAR	5.95	1.53	3.99	1.43	−0.15	3.56	0.32
HAR1	5.72	1.85	4.25	1.42	0.12	2.81	0.32
HAR2	6.27	1.59	4.18	1.42	0.04	1.07	0.32

The RMSE of BREB was 1.31, the RMSE for combination models ranged from 1.38 to 1.58 mm day^−1^ and averaged 1.47 mm day^−1^, the RMSE for radiation-based models ranged from 1.19 to 1.55 mm day^−1^ and averaged 1.40 mm day^−1^, and the RMSE for temperature-based models ranged from 1.42 to 1.43 mm day^−1^ and averaged 1.42 mm day^−1^ (Table 4). Based on the RMSE values, the performance of the fourteen evapotranspiration models followed the order: DK (1.19) > PT (1.22)> Makkink (1967) (1.28) > BREB (1.31) > Pen-63 (1.38) > HAR1 (1.42) > HAR2 (1.42)> HAR (1.43) > FAO-24 Pen (1.46) > Makkink (1.47) > IRMAK1 (1.54) > Makkink (1957) (1.55) > IRMAK2 (1.55) > PM-56 (1.58). Evidently, the best DK was 25% more accurate than the poorest (FAO-56). Overall, for the growing season period, the BREB yielded the best performance, followed by the radiation-based, temperature-based, and combination models.

### Comparison of Daily Evapotranspiration Between Reference Evapotranspiration Models and Lysimeter Measurements During the Nongrowing Season

**Figure 6:**
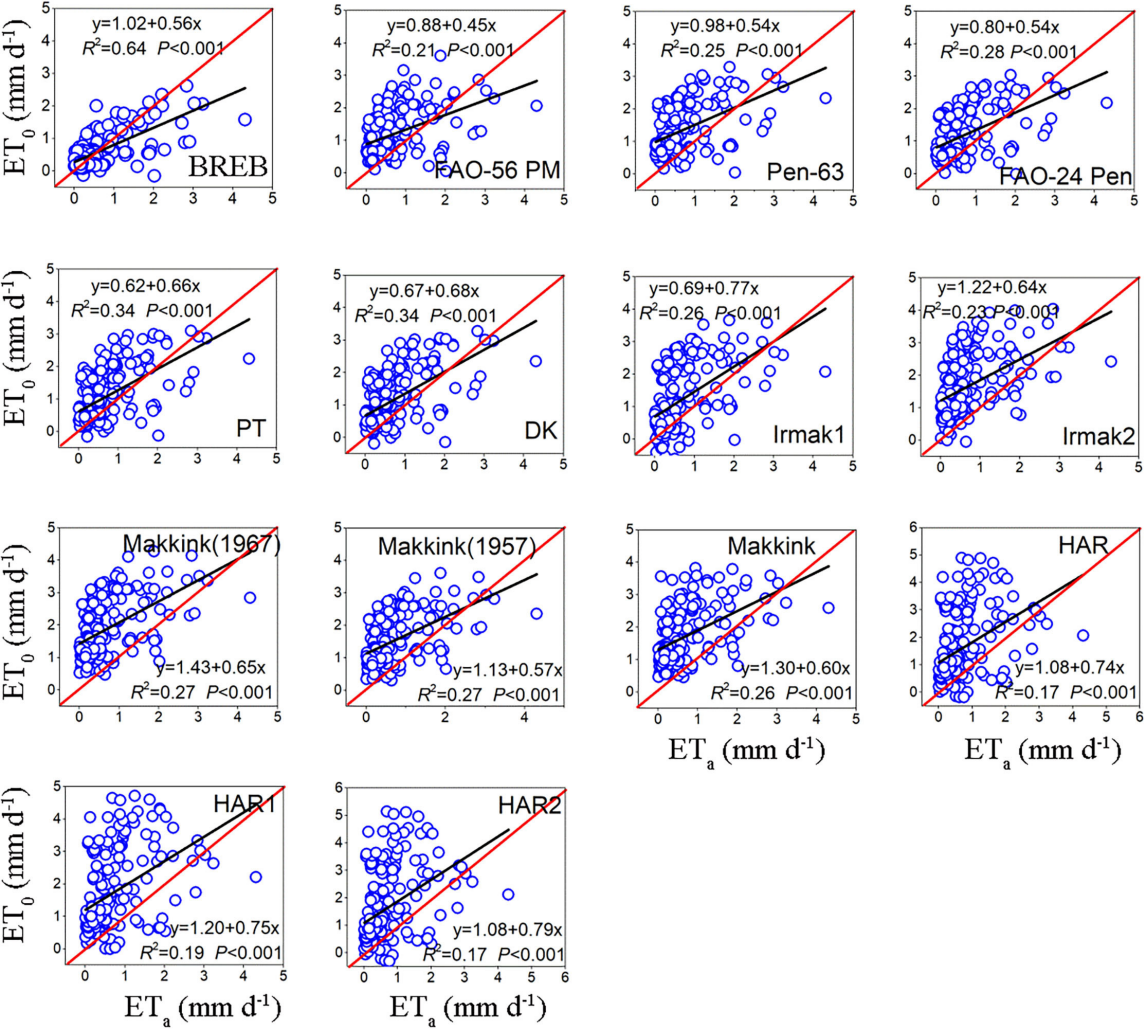
The relationship between daily ET_0_ of model estimates and ET_a_ of lysimeter measurements during the nongrowing season (data points = 143).

During the nongrowing season, the daily ET_0_ calculated by the fourteen evapotranspiration equations was also significantly correlated with the lysimeter measurements ET_a_ (*P* < 0.01), but with lower coefficients of determination (*R*^2^) ranging from 0.17 to 0.64 (Figure 6, Table 5). Of the combination models, FAO-24 Pen obtained the highest *R*^2^ (0.28), followed by Pen-63 (0.25) and PM-56 (0.21). Of the radiation-based models, PT and DK obtained the highest *R*^2^ (0.34), followed by Makkink (1967) (0.27), Makkink (1957) (0.27), Makkink (0.26), IRMAK1 (0.26), and IRMAK2 (0.23). Among the temperature-based models, HAR had the highest *R*^2^-value (0.19). Interestingly, all models (except BREB) generally overestimated the ET_a_ values measured by lysimeter during the nongrowing season, with MBEs ranging from 0.40 to 1.20 mm day^−1^ and averaging 0.78 mm day^−1^; Makkink (1967) yielded the largest underestimate (by 185.83%) and PT the minimum underestimate (by 61.06%) (Table 5).

**Table 5:** Summary of statistics for daily ET_0_ between lysimeter measurements and model estimates during the nongrowing season (data points = 204).

**Model**	**Values of ET**_**0**_ **(mm d**^**−1**^**)**			**Difference in ET**_**0**_			
	**Max**	**Min**	**Mean**	**RMSE (mm d^**−1**^)**	**MAE (mm d^**−1**^)**	**PE (%)**	***R*** **^2^**
Measured	4.31	0.01	0.65				
BREB	2.62	−0.16	0.62	0.52	−0.03	−5.03	0.64
FAO-56PM	3.60	0.06	1.17	0.90	0.53	81.21	0.21
Pen-63	3.29	0.04	1.33	1.00	0.68	104.73	0.25
FAO-24 Pen	3.03	−0.02	1.15	0.86	0.50	77.72	0.28
PT	3.11	−0.17	1.04	0.80	0.40	61.06	0.34
DK	3.28	−0.19	1.11	0.84	0.46	71.43	0.34
Makkink	3.82	0.45	1.69	1.29	1.04	160.58	0.26
Makkink(1967)	4.28	0.47	1.85	1.44	1.20	185.83	0.27
Makkink(1957)	3.61	0.29	1.49	1.11	0.85	130.57	0.27
IRMAK1	3.66	−0.83	1.19	1.07	0.54	83.42	0.26
IRMAK2	4.04	−0.05	1.63	1.30	0.98	151.95	0.23
HAR	4.89	−0.18	1.56	1.47	0.92	141.24	0.19
HAR1	4.71	−0.01	1.69	1.51	1.04	159.98	0.17
HAR2	5.14	−0.31	1.60	1.54	0.95	146.20	0.17

The RMSE of BREB was 0.52, the RMSE for combination models ranged from 0.86 to 1.00 mm day^−1^ and averaged 0.92 mm day^−1^, the RMSE for radiation-based models ranged from 0.80 to 1.44 mm day^−1^ and averaged 1.12 mm day^−1^, and the RMSE for temperature-based models ranged from 1.47 to 1.54 mm day^−1^ and averaged 1.50 mm day^−1^. Based on the RMSE, the ET_0_ model performance decreased in the following order: BREB (0.52) > PT (0.80) > DK (0.84) > FAO-24 Pen (0.86) > PM-56 (0.90) > Pen-63 (1.00) > IRMAK1 (1.07) > Makkink (1957) (1.11) > Makkink (1.29) > IRMAK2 (1.30)> Makkink (1967) (1.44) > HAR (1.47)>HAR1 (1.51) > HAR2 (1.54). Evidently, the best model was 66.24% more accurate than the poorest model (HAR2). Overall, for the nongrowing season period, the BREB yielded the best performance, followed by the combination, radiation-based, and temperature-based models.

### Comparison of Monthly Averaged Daily Evapotranspiration Between Reference Evapotranspiration Models and Lysimeter Measurements

**Figure 7:**
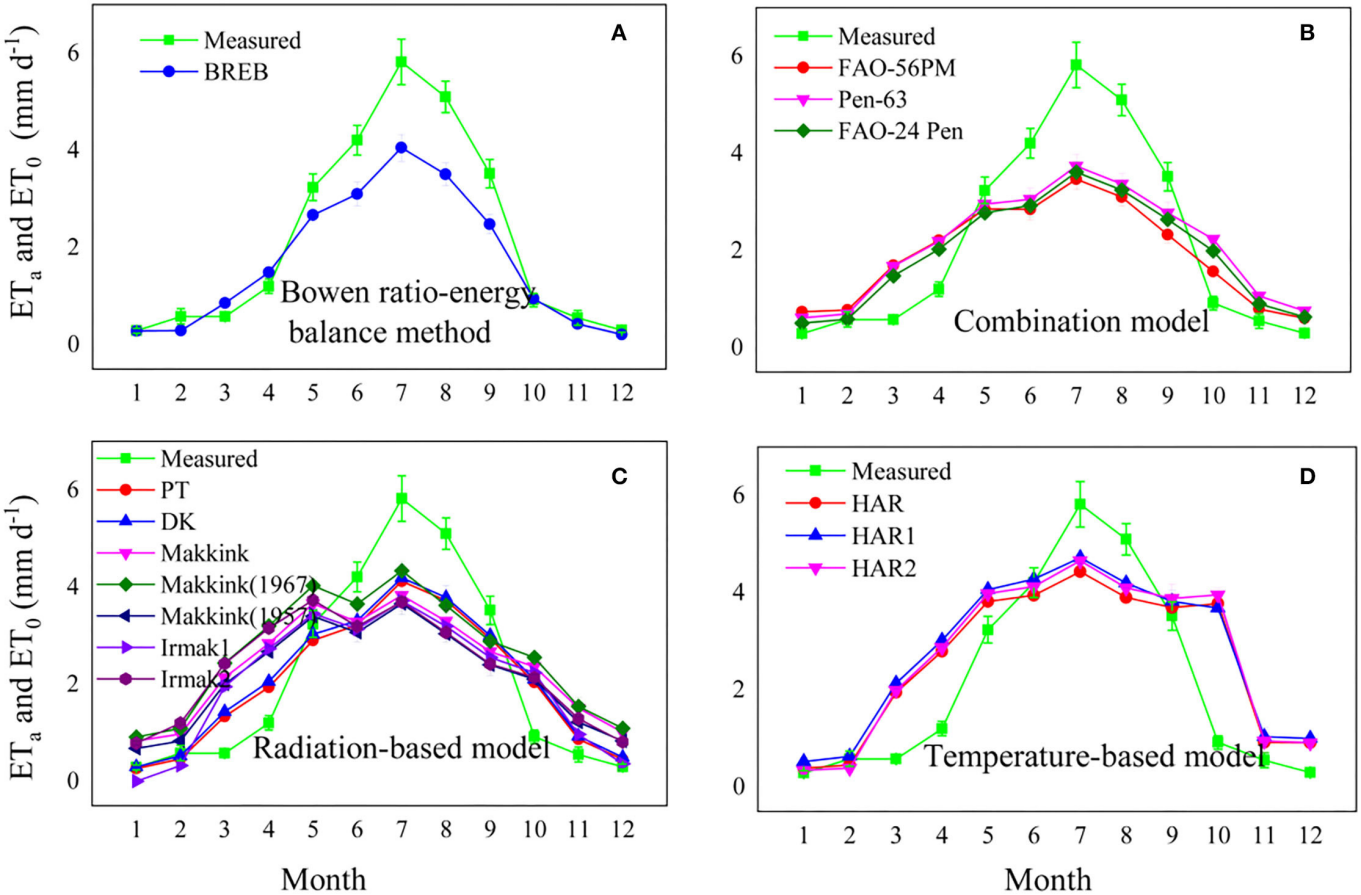
Comparison of monthly mean daily evapotranspiration between lysimeter measurements and model estimates, **(A)** Bowen ratio energy balance method, **(B)** combination model, radiation based model **(C)**, and temperature-based model **(D)**.

The fourteen evapotranspiration model estimations were consistent with the pattern observed in the lysimeter measurements (Figure 7), with a peak in July. As already noted, the combination models and BREB underestimated the measurements from May to September and overestimated those in the other months (Figures 7A,B). The radiation-based models underestimated the measurements from June to September and overestimated those in the other months (Figure 7C). However, the temperature-based models generally overestimated the measured evapotranspiration during most months (except for July and August) (Figure 7D). Overall, all models tended to underestimate the measured ET_a_ during the growing season (with larger evaporative demand) and overestimated ET_a_ during the nongrowing season (with reduced evaporative demand).

### Dominant Factors Affecting the Seasonal Variation in Lysimeter ET_a_ Measurements

**Figure 8:**
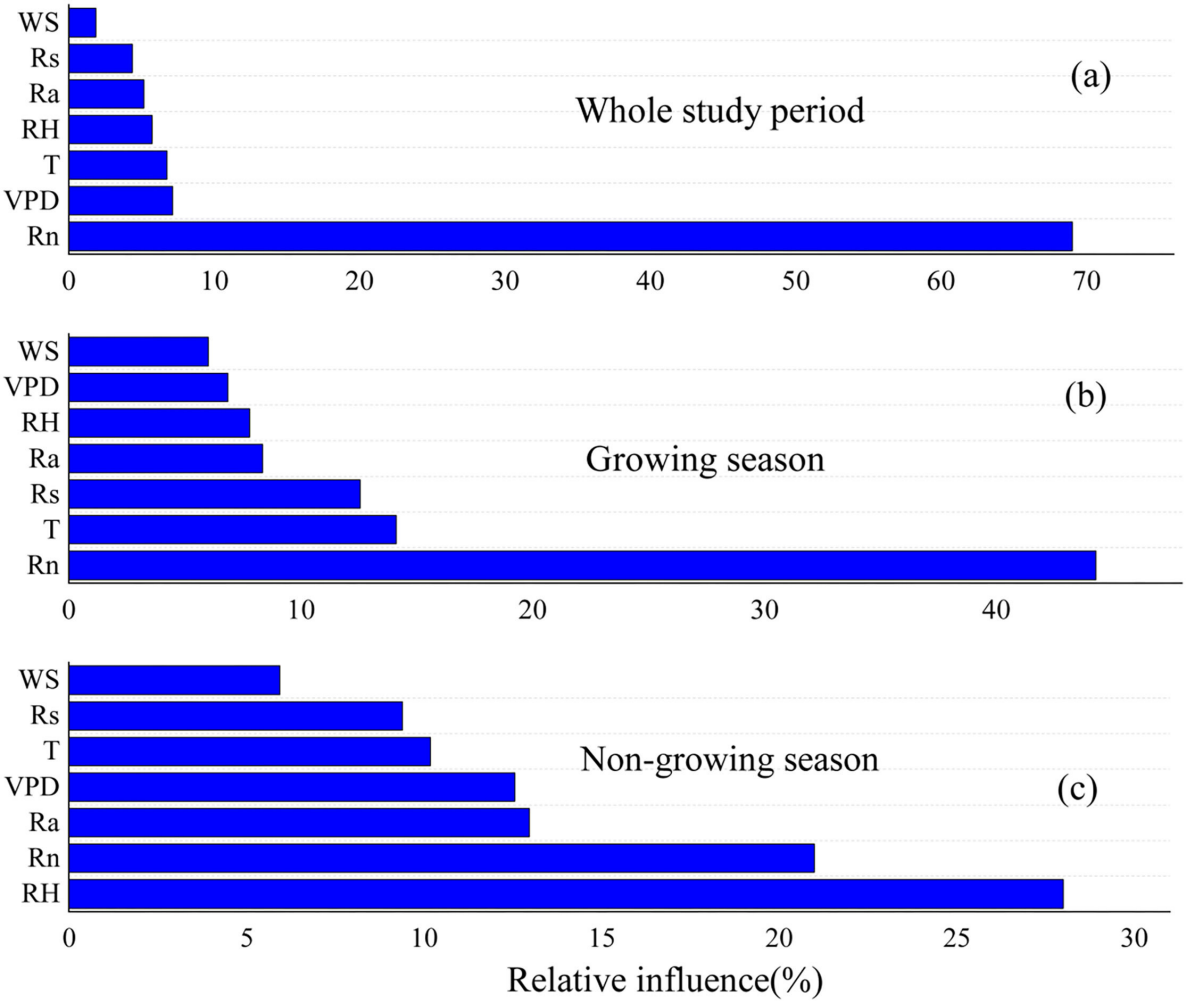
The relative influence of meteorological factors on ET_a_ during whole study period **(a)**, growing season **(b)**, and non-growing season **(c)**. WS, wind speed; TD, the difference value between maximum air temperature and the minimum air temperature; *G*, soil heat flux; R_a_, extraterrestrial solar radiation; R_s_, total radiation; VPD, vapor pressure deficit; RH, relatively humid; *T*, mean air temperature; *R*_n_, net radiation.

The BRT model indicated that *R*_n_ was the dominant factor controlling the seasonal variation in measured ET_a_ throughout the study period, accounting for 69.02% of the total variability, followed by VPD (7.13%), *T* (6.75%), RH (5.73%), *R*_a_ (5.15%), *R*_s_ (4.33%), and WS (1.85%) (Figure 8a). During the growing season, R_n_ remained the main control of seasonal variation in measured ET_a_ (Figure 8b), accounting for 44.30% of the total variability, followed by *T* (14.12%), *R*_s_ (12.56%), *R*_a_ (8.34%), RH (7.80%), VPD (6.87%), and WS (6.02%). However, the seasonal variation in the measured ET_a_ in the nongrowing season was dominated by RH (Figure 8c), accounting for 27.99% of the total variability, followed by *R*_n_ (20.99%), *R*_a_ (12.96%), VPD (12.56%), *T* (10.18%), *R*_s_ (9.39), and WS (5.94%) (Figure 8).

## Discussion

### Performance Comparison of Combination Models Against Lysimeter Measurements

Given that the evapotranspiration in our study was not limited by soil water conditions and the plant height was 8–12 cm, actively growing, and completely shading the ground, which is close to the definition of ET_0_, we thus assumed that ET_a_ was comparable to ET_0_ during the growing season, with the aim of selecting the best fit model over a humid alpine meadow on the northeastern QTP to estimate ET_0_. Previous studies have shown that the Penman family models are generally the most accurate when evaluating ET_0_ across various climate scenarios and regions (Liu et al., 2017). Of the Penman models for ET_0_, the PM-56 has been considered the standard equation for estimating evapotranspiration (Allen et al., 1998). For instance, Yoder et al. (2005) found that PM-56 displayed the best performance in the humid southeast United States. López-Urrea et al. (2006) tested seven evapotranspiration equations using lysimeter observations in a semiarid climate and found that the PM-56 equation was the most precise method compared with other evapotranspiration equations. Unlike previous studies, the PM-56 model was not the best in our study; we found that Pen-63 and FAO-24 Pen were more accurate (Pen-63 and FAO-24 Pen had smaller RMSE than PM-56 throughout the study period). Similar results have been reported in other studies (Berengena and Gavilán, 2005; Martel et al., 2018). A more recent study also reported the poor performance of PM-56 when compared with data from 20 FLUXNET towers (Ershadi et al., 2014). These results suggest that PM-56 might not be the only standard model for evaluating ET_0_ because it did not yield better accuracy than the other Penman models. Given the better performance of Pen-63 and FAO-24 than PM-56 in this study, we may apply old Penman family models to our study region, especially considering that the PM-56 requires many meteorological inputs, which limits its use in areas with sparse data, especially in harsh environments (Tabari et al., 2012). Overall, the poor performance of PM-56 may be attributed to the higher aerodynamic resistance (*r*_s_), and there is increasing evidence indicating that PM-56 underestimation is related to the fixed *r*_s_ = 70 s m^−1^ in the equation, which may be too large (Liu et al., 2017). This result was also confirmed by our results that the values of RMSE increased from 1.22 to 1.29 mm day^−1^ as *r*_s_ changed from 20 to 60 s m^−1^, and the RMSE was nearly unchanged (from 1.12 to 1.13 mm day^−1^) when *r*_s_ varied between 0 and 20 s m^−1^ (Figure 4). Therefore, reducing the value of *r*_s_ from 70 to 0–20 s m^−1^ can improve daily PM-56 estimates. Other studies also found that *r*_s_ should be a variable rather than a fixed value (Allen et al., 2006; Liu et al., 2017). For instance, *r*_s_ should be smaller when the result is underestimated and should be larger when it is overestimated (Ventura et al., 1999).

### Performance Comparison of Radiation and Temperature Models Against Lysimeter Measurements

For the performance comparison of radiation models against lysimeter measurements, we found that the PT models yielded the best performance of the radiation-based models, which was consistent with a previous study conducted in humid areas where the PT method yielded a good accuracy estimate for ET_0_ (Ershadi et al., 2014). There is increasing evidence indicating that the input parameters are the dominant factors affecting their performance (Lang et al., 2017), and we conclude that the better performance of the PT models might be associated with the most important meteorological factors affecting ET, such as *R*_n_, which is supported by our results (Figure 8). Compared with the PT method, other radiation models that use *R*_s_ as the main driving variable may overestimate ET_0_ due to the reduction in *R*_s_ through atmospheric reflection in this region (Zhang et al., 2018). Furthermore, each model was developed based on its specific underlying surface and climatic conditions. For instance, the PT model was established in a humid climate condition, which is suitable for a humid alpine meadow in the northeastern QTP. Most importantly, the PT models require fewer meteorological inputs than the combination models. However, given that the ET_a_ was mainly limited by available radiation (i.e., *R*_n_-G), the structures of the PT and DK models both included available radiation items. Combining these factors, we recommend the PT and DK models for use in humid alpine meadows in the northeastern QTP, especially when considering the difficulty in obtaining evapotranspiration in this harsh climate.

For the performance comparison of temperature models against lysimeter measurements, a previous study reported that the Hargreaves equation is one of the simplest empirical methods used for ET_0_ estimation because of its lower meteorological data input, including some meteorological data required in the standard PM-56 model (Jensen et al., 1990). To further select the best Hargreaves version equation, we compared the performance of the original (HAR) and two modified versions (HAR1 and HAR2) and found that the original HAR model had the lowest error (RMSE = 1.47 mm day^−1^, MAE = 0.40 mm day^−1^, and PE = 17.37%), which was consistent with previous studies conducted in humid regions (Tabari, 2010) but contrasted to studies conducted in arid regions in which the modified Hargreaves equation displayed a more accurate estimation of evapotranspiration (Ravazzani et al., 2012). Overall, the temperature models displayed poor performance compared with radiation models because the Hargreaves method was established in semiarid areas (Tabari, 2010). Furthermore, the structure of temperature models missing the most important parameter (i.e., *R*_n_) results in poor performance compared with radiation models. Therefore, a local calibration is required to improve the Hargreaves method accuracy in nonarid regions.

### Performance Comparison of All Models

By comparing the four model types, we found that the BREB yielded the best performance, followed by the combination, radiation-based, and temperature-based models (Table 3). Overall, most radiation-based models underestimated the measured ET_a_ throughout the study period, whereas the temperature-based models tended to overestimate ET_a_. This was consistent with previous studies in which the Makkink and PT models generally underestimated ET_a_ (Priestley and Taylor, 1972; Xu and Singh, 2002), while the Hargreaves equations often overestimated ET_a_ in cold-humid conditions and required local calibration (Berti et al., 2014). Given that our study region was a humid alpine meadow, ET_a_ tended to be overestimated. An alternative explanation for the poor Hargreaves model performance in humid regions may be that the Hargreaves method was established in semiarid areas (Tabari, 2010), and the *R*_a_ parameter used in the Hargreaves model structure was based on the maximum possible radiation value and does not consider the atmospheric transmissivity. However, the atmospheric transmissivity in humid regions is affected by many factors, such as atmospheric moisture; thus, the solar radiation reaching the surface is significantly reduced because of the high atmospheric moisture content (Temesgen et al., 1999), resulting in the overestimation of solar radiation, ultimately leading to an overestimation of evapotranspiration using the Hargreaves method.

Furthermore, there were common features in all four groups of models. All the models tended to underestimate the measured ET_a_ during the growing season (with larger evaporative demand) and overestimated ET_a_ during the nongrowing season (with reduced evaporative demand), which was consistent with a previous study conducted in a semiarid region (Liu et al., 2017). The underestimated measured ET_a_ during the growing season may be related to the ET_a_ in alpine meadows under strongly energy-limited conditions rather than soil water content during the growing season; thus, higher solar radiation could lead to a higher ET_a_ during the growing season (Zhang et al., 2018). Therefore, both the Hargreaves equations and other models require further local or regional calibration before being applied to a given region (Xu and Singh, 2002). It should also be noted that the data used in this study were obtained from 1 year and a single weather station, which may be insufficient to represent the whole humid climate or the alpine ecosystem but represent only a humid alpine meadow in the northeastern QTP. Thus, a longer period and more lysimeter systems should be used in the alpine ecosystem in the future to obtain more accurate estimates of evapotranspiration over humid alpine meadows in the northeastern QTP.

## Conclusion

This study is the first to document information on the comparison of fourteen evapotranspiration models against lysimeter measurements in a humid alpine meadow, northeastern Qinghai-Tibetan Plateau, and we found that the BREB method performed the best, followed by combination models, radiation-based models, and temperature-based models. Furthermore, all models tended to underestimate ET_a_ during the growing season and overestimate ET_a_ during the nongrowing season, suggesting that these models should be calibrated or modified by local lysimeter data when extrapolated to other regions. Besides, the 1963 Penman and FAO-24 Penman models demonstrated better performances than recommended FAO-56 PM, suggesting that older Penman equations may superior to the standard FAO-56 PM model. Given the outstanding performance of Priestley–Taylor model owing to its most important factors affecting ET_a_ (*R*_n_), which require few meteorological inputs, we thus recommend that these two models can be used in the humid alpine meadow on the northeastern Qinghai-Tibetan Plateau. Our result could provide new insights for the accurate assessment of evapotranspiration in the alpine ecosystem.

## Data Availability Statement

The original contributions presented in the study are included in the article/supplementary material, further inquiries can be directed to the corresponding authors.

## Author Contributions

LD and XG performed the research, analyzed data, and wrote the manuscript. RF, ZZ, ZH, and YD analyzed data. GC conceived the study. All authors contributed to the article and approved the submitted version.

## Funding

This study was supported by the National Natural Science Foundation of China (41730752) and the Natural Science Foundation of Qinghai (2021-HZ-811). Start-up funding from Hainan University (KYQD(ZR)-22085). Start-up funding from Hainan University (KYQD(ZR)-22085).

## Conflict of Interest

The authors declare that the research was conducted in the absence of any commercial or financial relationships that could be construed as a potential conflict of interest.

## Publisher's Note

All claims expressed in this article are solely those of the authors and do not necessarily represent those of their affiliated organizations, or those of the publisher, the editors and the reviewers. Any product that may be evaluated in this article, or claim that may be made by its manufacturer, is not guaranteed or endorsed by the publisher.
