# Corrigendum: Comparison of 14 reference evapotranspiration with lysimeter measurements at a site in the humid alpine meadow, northeastern Qinghai-Tibetan Plateau

**DOI:** 10.3389/fpls.2022.945254

**Published:** 2022-07-13

**Authors:** Licong Dai, Ruiyu Fu, Zhihui Zhao, Xiaowei Guo, Yangong Du, Zhongmin Hu, Guangmin Cao

**Affiliations:** ^1^College of Ecology and Environment, Hainan University, Haikou, China; ^2^Hainan Academy of Forestry, Haikou, China; ^3^Qinghai Provincial Key Laboratory of Restoration Ecology for Cold Region, Northwest Institute of Plateau Biology, Chinese Academy of Sciences, Xining, China

**Keywords:** reference evapotranspiration, alpine meadow, lysimeter measurement, combination models, radiation-based models, temperature-based models

In the original article, there was a mistake in Figure 1 as published. The corrected Figure 1 appears below.

**Figure 1:**
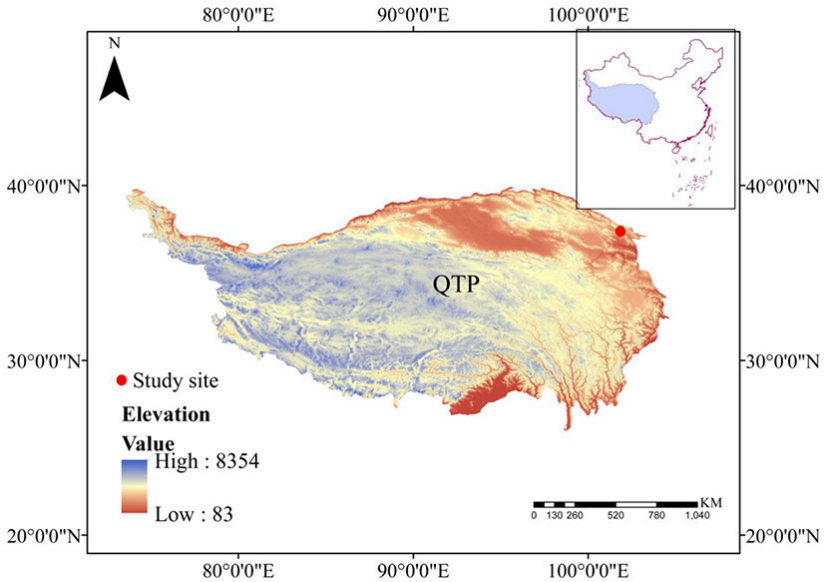
The location of study area in our study.

The authors apologize for this error and state that this does not change the scientific conclusions of the article in any way. The original article has been updated.

## Publisher's note

All claims expressed in this article are solely those of the authors and do not necessarily represent those of their affiliated organizations, or those of the publisher, the editors and the reviewers. Any product that may be evaluated in this article, or claim that may be made by its manufacturer, is not guaranteed or endorsed by the publisher.

